# Legal Infoveillance of Unlicensed Medical Practices in South Korea Through Criminal Court Decisions Using Machine Learning: Retrospective Observational Study

**DOI:** 10.2196/92591

**Published:** 2026-06-15

**Authors:** Min Ji Kim, Min Choi

**Affiliations:** 1 Department of Medical Humanities and Ethics College of Medicine Hanyang University Seoul Republic of Korea; 2 LBox Corporation Seoul Republic of Korea

**Keywords:** unlicensed medical practice, machine learning, natural language processing, patient safety, scope of practice, legal infoveillance, artificial intelligence, AI, Republic of Korea

## Abstract

**Background:**

Unlicensed medical practices (UMPs) pose a substantial threat to patient safety and public health, but their clandestine nature makes them difficult to monitor through conventional surveillance systems. Legal epidemiology offers a framework for using judicial data to study hidden health-related misconduct, and machine learning (ML) may help convert unstructured legal texts into analyzable public health information.

**Objective:**

This study aimed to characterize prosecuted UMP cases in South Korea using a legal infoveillance framework and evaluate the utility of ML-assisted extraction from criminal court decisions for public health surveillance.

**Methods:**

We conducted a retrospective observational study of 1532 criminal court decisions involving UMP-related convictions in South Korea between 2005 and 2023. Using an ML-assisted extraction pipeline with human-in-the-loop verification, we transformed unstructured judicial texts into structured legal and medical variables. Analyses were conducted at the case, charge, defendant category, and legal ruling levels. In addition to descriptive analyses, we performed exploratory inferential analyses to examine factors associated with legal rulings and professionals’ involvement.

**Results:**

Of 1718 charge entries, 987 (57.5%) were related to Article 5 of the Act on Special Measures for the Control of Public Health Crimes, and 731 (42.5%) were related to Article 27(1) of the Medical Service Act. Profit motive was coded in 91.6% (1404/1532) of the cases. At the legal ruling level (n=2004 entries), suspended sentences, meaning sentences whose execution was conditionally suspended under Korean criminal law, were the most common outcome (1261/2004, 62.9%), followed by fines (421/2004, 21%) and imprisonment without suspension (209/2004, 10.4%). Of 1716 defendant category entries, ordinary persons accounted for 1294 (75.4%), health care professionals accounted for 264 (15.4%), and health care providers accounted for 158 (9.2%). Physicians were the largest subgroup among health care professionals. In exploratory multinomial models, licensed personnel–only and mixed ordinary person and licensed personnel cases were more likely than ordinary person–only cases to result in fines or imprisonment without suspension rather than suspended sentences. Secondary exploratory analyses also suggested distinctive patterns of professional involvement and possible scope-of-practice or delegation-related boundary violations.

**Conclusions:**

ML-assisted analysis of criminal court decisions can serve as a useful supplementary surveillance method for hidden UMPs. In South Korea, prosecuted UMPs were predominantly profit driven and involved both ordinary persons and licensed personnel. The findings support closer monitoring of scope-of-practice and delegation-related violations and demonstrate the value of judicial records as a source of public health intelligence.

## Introduction

The World Health Organization identifies unsafe care as a major global health challenge, with millions of patients harmed annually due to inadequate safety standards and unqualified practitioners [1]. One persistent but difficult-to-measure contributor is medical or quasi-medical practices by persons acting outside legally defined professional boundaries. Unlicensed medical practices (UMPs) include both practices performed by persons with no health-related license and practices performed by licensed personnel who exceed, improperly delegate, or otherwise violate their lawful scope of practice [2].

UMPs are difficult to study using conventional surveillance tools because they are inherently concealed. Administrative health data, claims systems, and routine public health reporting rarely capture such activity directly. In South Korea, as universal health coverage coexists with the strict licensing regime, UMPs may persist not because care is unavailable [3] but because commercially motivated noncompliance and legal ambiguity regarding the boundaries of “medical practice” create opportunities for underground or improperly delegated services [4].

In South Korea, the Medical Service Act strictly prohibits UMPs under Article 27(1) to protect patient safety [5]. Additionally, Article 5 of the Act on Special Measures for the Control of Public Health Crimes imposes severe penalties, including imprisonment, on nonprofessionals engaging in UMPs for profit [6]. However, the statutory provisions do not explicitly define “medical practice,” leaving courts with wide discretion in interpreting the boundaries given potential risks to public health. Furthermore, these practices often lead to clinical negligence, compromising both patient safety and the integrity of the health care system. Therefore, establishing a stringent regulatory regime based on accurate situational awareness is crucial [7,8].

Despite its significant threat to public health, the inherently clandestine nature of UMPs makes them difficult to quantify [9]. Previous studies have been limited to regulatory reviews [10,11] or localized cross-sectional surveys [12], leaving a critical empirical gap regarding the actual prevalence and distinct characteristics of these offenses. Therefore, investigating the current status of UMPs using objective, large-scale data is essential to provide foundational evidence for regulatory policymaking.

South Korea provides a strategically informative setting for this approach because court decisions are highly digitized [13,14] and can be assembled at scale for computational analysis [15]. Recent work in legal natural language processing has made it increasingly feasible to extract structured variables from unstructured judicial text and analyze legal outcomes systematically [16,17]. Within the framework of legal epidemiology, which treats law and legal processes as measurable public health phenomena [18], we propose the term “legal infoveillance” to denote the systematic use of legal and judicial records as a source of surveillance for population health questions. This differs from conventional digital infoveillance, which relies primarily on internet-based or digitally generated information streams [19,20], because legal infoveillance relies on formal case records reflecting detected, investigated, prosecuted, and adjudicated events. Furthermore, while legal epidemiology examines the law as a factor in the distribution and prevention of disease and digital infoveillance monitors public health trends using internet-based behavioral data, our framework synthesizes and extends these domains. By integrating principles of legal epidemiology with recent advancements in artificial intelligence [20], we propose a scalable solution to this data bottleneck. Machine learning (ML) techniques can now process massive volumes of legal texts to predict outcomes and understand the factors influencing judicial decisions [16,17]. Applying these technologies to UMP precedents allows for a novel form of public health surveillance, turning retrospective legal data into actionable public health intelligence [18].

This study aimed to assess the status and characteristics of UMPs by analyzing a comprehensive dataset of criminal precedents using ML techniques. To our knowledge, this is the first study to use automated legal text analysis to generate evidence-based knowledge for global UMP policymaking and regulatory design.

## Methods

### Study Design and Data Collection

This study was a retrospective observational study of criminal court decisions involving UMP-related convictions in South Korea between 2005 and 2023. We identified closed criminal cases in which at least one defendant was convicted of an offense related to UMPs. The dataset was filtered from a comprehensive corpus of over 3 million court precedents collected by LBox, a legal technology company in South Korea [21]. We selected 2005 as the starting year because systemic indexing in the source corpus began in that year, allowing for consistent retrieval and longitudinal comparison across the study period [13,15]. This dataset serves as a proxy for the detected portion of UMPs, offering a rare window into the types of practices and defendants capture through regulatory oversight. This manuscript was reported with reference to the STROBE (Strengthening the Reporting of Observational Studies in Epidemiology) statement [22], and the completed checklist is provided in Multimedia Appendix 1.

### Data Processing

Processing raw legal data posed significant challenges because court decisions are often stored as unstructured images or PDFs. To address this, we used a specialized data processing pipeline [15]. First, a rule-based layout classifier distinguished text-only pages from those containing tables or figures. Then, texts were extracted using LBox’s custom rule-based parser for PDFs and an optical character recognition engine for image files [23]. Third, to extract variables from the unstructured text bundles, a custom-made parser based on bidirectional encoder representations from transformers [24] segmented the texts into functional components, such as facts, legal rulings, reasons for sentencing, and conclusions. This methodology aligns with emerging standards in legal text mining for public health, enabling the conversion of complex narrative data into structured epidemiological variables.

### ML-Assisted Extraction and Human Verification

We used an enhanced proprietary version of the end-to-end Information Extractor for Statistical Legal Analysis [25], an end-to-end neural system based on a domain-adapted mT5-Large architecture [26], for automated information extraction. The foundational model has been validated, achieving an average *F*_1_-score of 93.1 across Korean legal information extraction tasks [25].

**Figure 1:**
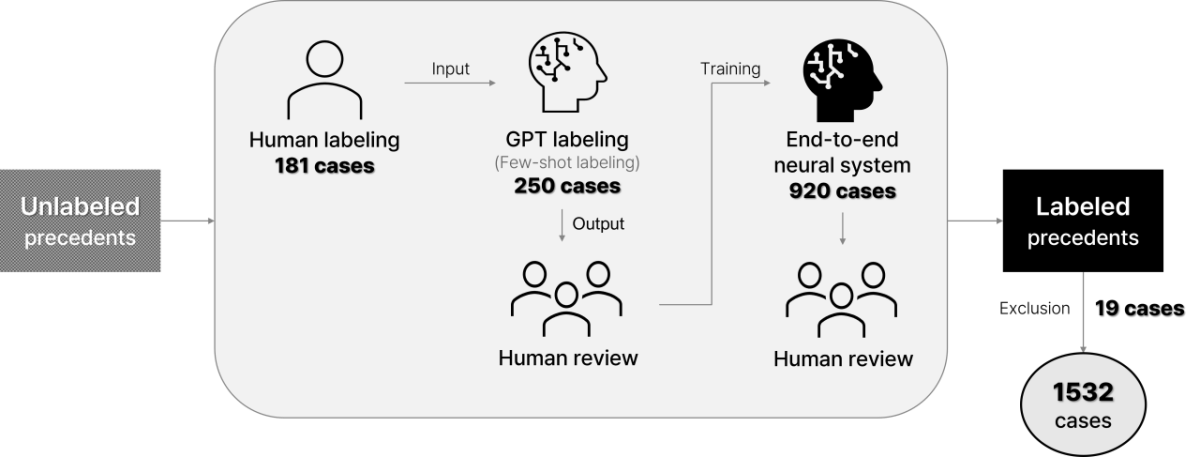
Machine learning–assisted information extraction and human-in-the-loop labeling workflow. GPT: generative pretrained transformer.

Given the high-stakes nature of legal and health data, we addressed the “black box” problem of artificial intelligence by implementing a rigorous human-in-the-loop validation process. Initially, 181 cases were labeled by the researchers to create the gold-standard set, which provided the few-shot examples for GPT-4 (OpenAI) [27] to generate labels for an additional 250 cases [28]. Second, these model-generated labels were thoroughly reviewed and corrected by 3 professional labelers, yielding a verified dataset of 631 cases. Third, this corrected dataset was used to fine-tune the Information Extractor for Statistical Legal Analysis–based system, which subsequently generated variable values autoregressively for the remaining cases. In total, 920 cases were labeled by the fine-tuned system and verified by the labelers. Cases involving complex compound charges (eg, UMPs combined with drug-related charges) were excluded (n=19) to ensure reliability. Finally, 1532 unique labeled cases were included in the final analysis (Figure 1).

### Analytic Units and Variable Definitions

As a single judicial decision could involve multiple charges, defendants, and legal rulings, counts across analytic levels were not directly comparable. Therefore, all percentages are reported with their explicit denominators. Analyses were conducted at 4 levels: cases (n=1532), charges (n=1718), defendants (n=1716), and legal rulings (n=2004).

Variables were defined through close collaboration between experts in computer science and medical law. For practitioner classification, we used 3 operational categories: health care professionals, health care providers, and ordinary persons. Health care professionals were defined as physicians, dentists, doctors of Korean medicine, midwives, and registered nurses under the Medical Service Act of South Korea [5]. Health care providers were defined as other licensed or certified health care–related personnel, such as nurse aides, medical technologists, and physical therapists. Finally, we defined ordinary persons as defendants without a health care–related professional license. Legal rulings were classified as imprisonment without suspension, fines, suspended sentences, suspension of imposition of sentence, acquittal, or other. Sentencing factors (eg, settlement with victims and self-reflection) were categorized according to the guidelines of the Sentencing Commission of South Korea [29].

### Data Analysis

The variables were labeled and classified into legal and medical sections for each case. Before labeling the variables, ontologies were built for the individual sections, and a labeling page was set up using Label Studio (HumanSignal) [30]. To ensure the validity of the results, the researchers independently labeled the variables for each case (181 samples) and then cross-checked them. The section on legal facts and rulings was labeled by a PhD in Engineering at LBox (MC), which provides a cloud-based service to support the digitization of legal practices by processing court documents and records in South Korea [21]. A PhD in Medical Law and Ethics (MJK) verified the medical sections to ensure accuracy.

Statistical analyses were performed using SPSS (version 27.0; IBM Corp) and SAS (version 9.4; SAS Institute Inc). We first conducted descriptive analyses at the case, charge, defendant category, and ruling entry levels. We then examined annual case counts from 2005 to 2023 to visualize the temporal distribution of UMP cases prosecuted. Because the dataset was conditioned on prosecuted cases with at least one UMP-related conviction, inferential analyses were framed as exploratory models of sentencing patterns among detected, prosecuted, and adjudicated cases rather than as estimates of underlying UMP prevalence, probability of offending, probability of conviction, or guilt. For the primary inferential analysis, we fit a multinomial logistic regression using the principal legal ruling recorded in each case as the dependent variable. Suspended sentence was used as the reference, and suspension of imposition of sentence, acquittal, and other rare outcomes were collapsed into a single “others” category due to small cell counts. Predictors included case composition (ordinary person only, licensed personnel only, mixed ordinary person and licensed personnel, or uncoded), involvement in the Act on Special Measures for the Control of Public Health Crimes charges, profit motive, and practice type. As secondary analyses, we fit logistic models of professionals’ involvement among category-coded cases and of health care providers’ coinvolvement among licensed personnel–involved cases as an exploratory proxy for delegation- or boundary-related scenarios.

### Ethical Considerations

The joint institutional review board designated by the Ministry of Health and Welfare of South Korea confirmed that the study protocol met the criteria for exemption from regular review because this research was based on literature that was publicly accessible by any individual and owing to the absence of direct or indirect involvement of human materials, animal experiments, or pathogens (P01-202406-01-025).

## Results

### Overview of UMP Cases

**Figure 2:**
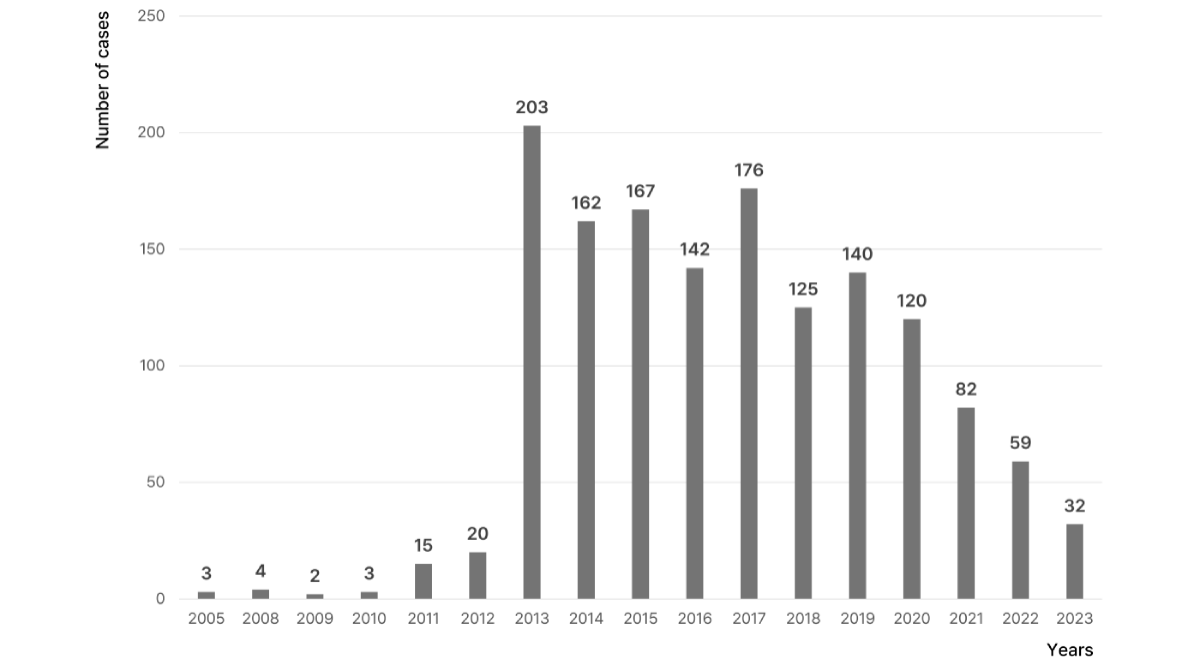
Annual distribution of 1532 prosecuted unlicensed medical practice cases identified from Korean criminal court decisions (2005-2023).

A total of 1532 criminal cases were analyzed. The annual distribution of these cases from 2005 to 2023 is illustrated in Figure 2, showing the longitudinal trends in UMP prosecutions. Annual case counts remained low until 2011, increased sharply from 2012, peaked at 203 cases in 2013, remained high until 2019, and steadily declined to 32 cases by 2023.

### Legal Characteristics and Rulings

All cases contained 1718 charge entries, of which 987 (57.5%) were related to violations of Article 5 of the Act on Special Measures for the Control of Public Health Crimes, whereas 731 (42.5%) were related to violations of Article 27(1) of the Medical Service Act. Profit motive was coded in 91.6% (1404/1532) of cases, indicating that the vast majority of prosecuted UMP cases were commercially motivated. At the legal ruling level (n=2004), suspended sentences were most common (1261/2004, 62.9%), followed by fines (421/2004, 21%), imprisonment without suspension (209/2004, 10.4%), and acquittal (57/2004, 2.8%). Analysis of sentencing factors revealed that payment or deposit for victims was recorded in 72.4% (63/87) of cases, settlements with victims were recorded in 75.4% (101/134) of cases, and self-reflection was recorded in 95.6% (586/613) of cases. Prior similar-offense counts were coded for 26.9% (412/1532) of cases, with a mean of 0.8 prior similar offenses per coded case (SD 1.2; range 0-12), and prior different-offense counts were coded for 11.3% (173/1532) of cases, with a mean of 0.3 prior different offenses per coded case (SD 0.6; range 0-5; Table 1).

**Table 1:** Legal characteristics and sentencing factors in prosecuted unlicensed medical practice cases identified from Korean criminal court decisions (2005-2023) in a retrospective observational study.

Category and variable				Values
**Charges (n=1718), n (%)**				
	Violation of Article 5 of the Act on Special Measures for the Control of Public Health Crimes			987 (57.5)
	Violation of Article 27(1) of the Medical Service Act			731 (42.5)
**For-profit motive (n=1532), n (%)**				
	Yes			1404 (91.6)
	No			128 (8.4)
**Legal ruling (n=2004), n (%)**				
	Suspended sentences			1261 (62.9)
	Fine			421 (21)
	Imprisonment without suspension			209 (10.4)
	Acquittal			57 (2.8)
	Suspension of imposition of sentence			51 (2.5)
	Others			5 (0.2)
**Sentencing factors**				
	**Payment or deposit for victims (n=87), n (%)**			
		Done	63 (72.4)	
		Not done	24 (27.6)	
	**Settlement with victims (n=134), n (%)**			
		Done	101 (75.4)	
		Not done	33 (24.6)	
	**Self-reflection (n=613), n (%)**			
		Yes	586 (95.6)	
		No	27 (4.4)	
	**Criminal records, mean (SD; range)**			
		Prior similar offenses per coded cases (n=412)	0.8 (1.2; 0-12)	
		Prior different offenses per coded cases (n=173)	0.3 (0.6; 0-5)	

### Medical Characteristics and Defendant Profiles

Defendant category coding was available for 97.8% (1499/1532) of cases, yielding 1716 defendant category entries as some cases involved more than one defendant category. Ordinary persons accounted for 75.4% (1294/1716); whereas health care professionals accounted for 15.4% (264/1716); and health care providers accounted for 9.2% (158/1716), including nurse aides, medical technologists, or physical therapists. Among health care professionals, physicians were the most frequent offenders (166/1716, 9.7%), followed by doctors of Korean medicine (41/1716, 2.4%) and dentists (35/1716, 2%). At the case level (n=1532), general UMP was the most common practice type (1056/1532, 68.9%), followed by unlicensed Korean medical practice (302/1532, 19.7%) and unlicensed dental practice (175/1532, 11.4%). Notably, one case was double coded for both general unlicensed medical and Korean medical practice (Table 2).

**Table 2:** Medical characteristics, defendant categories, and practice types in prosecuted unlicensed medical practice cases identified from Korean criminal court decisions (2005-2023) reported by analytic unit.

Category and variable		Frequency, n (%)
**Defendant category (defendant entries; n=1716)**		
	Ordinary person	1294 (75.4)
	Health care provider	158 (9.2)
	Health care professional	264 (15.4)
	Physician	166 (9.7)
	Doctor of Korean medicine	41 (2.4)
	Dentist	35 (2)
	Registered nurse	22 (1.3)
**Type of practice (cases; n=1532)**		
	Unlicensed medical practice^a^	1056 (68.9)
	Unlicensed Korean medical practice	302 (19.7)
	Unlicensed dental practice	175 (11.4)

^a^The general unlicensed medical practice category included tattoo-related cases when the conduct was adjudicated as unlicensed medical practice under Korean law; however, these cases were not analyzed as a separate practice type category in the revised analyses.

### Sanctions by Defendant Category

The distribution of legal rulings varied by defendant category. In the cross-tabulation of legal rulings and defendant categories among 1499 category-coded cases, suspended sentences (1129/1294, 87.2%) were the most frequently reported among ordinary person–involved cases. In contrast, fines were most frequently observed among physician-involved cases (130/166, 78.3%), followed by dentist-involved cases (26/35, 74.3%) and doctor of Korean medicine–involved cases (31/41, 75.6%; Table 3).

**Table 3:** Case-level multiple-response cross-tabulation of legal rulings and defendant categories in prosecuted unlicensed medical practice cases identified from Korean criminal court decisions (2005-2023)^a^.

Defendant category			Cases, n/N (%)^b^		Imprisonment without suspension, n/N (%)^c^		Fine, n/N (%)^c^		Suspended sentences, n/N (%)^c^		Suspension of imposition of sentence, n/N (%)^c^		Acquittal, n/N (%)^c^		Others, n/N (%)^c^
**Health care professionals**															
	Physician	166/1499 (11.1)		36/166 (21.7)		130/166 (78.3)		90/166 (54.2)		24/166 (14.5)		17/166 (10.2)		1/166 (0.6)	
	Dentist	35/1499 (2.3)		4/35 (11.4)		26/35 (74.3)		15/35 (42.9)		2/35 (5.7)		4/35 (11.4)		0/35 (0)	
	Doctor of Korean medicine	41/1499 (2.7)		7/41 (17.1)		31/41 (75.6)		26/41 (63.4)		6/41 (14.6)		3/41 (7.3)		0/41 (0)	
	Registered nurse	22/1499 (1.5)		4/22 (18.2)		11/22 (50)		9/22 (40.9)		2/22 (9.1)		5/22 (22.7)		0/22 (0)	
Health care providers			158/1499 (10.5)		31/158 (19.6)		110/158 (69.6)		99/158 (62.7)		24/158 (15.2)		14/158 (8.9)		0/158(0)
Ordinary persons			1294/1499 (86.3)		167/1294 (12.9)		249/1294 (19.2)		1129/1294 (87.2)		20/1294 (1.5)		34/1294 (2.6)		5/1294 (0.4)
Total			1499/1499 (100)		201/1499 (13.4)		397/1499 (26.5)		1232/1499 (82.2)		49/1499 (3.3)		53/1499 (3.5)		5/1499 (0.3)

^a^Because a single case could involve multiple defendant categories and multiple legal rulings, counts across rows are non–mutually exclusive; therefore, row percentages may add up to more than 100.

^b^The column shows the number and percentage of total analyzed cases (n=1499) that involved at least one defendant in the specified category.

^c^Values constitute the number and percentage of cases in each defendant category.

### Exploratory Inferential Analysis of Legal Rulings

To address the determinants of legal rulings, we fit an exploratory multinomial logistic regression model. Compared with ordinary person–only cases, licensed personnel–only cases were more likely to result in fines (adjusted relative risk ratio [aRRR] 2.13, 95% CI 1.36-3.32) and imprisonment without suspension rather than suspended sentences (aRRR 1.79, 95% CI 1.02-3.16). Mixed ordinary person and licensed personnel cases showed even stronger associations with fines (aRRR 3.56, 95% CI 1.62-7.81) and imprisonment without suspension (aRRR 2.75, 95% CI 1.18-6.40). Act on Special Measures for the Control of Public Health Crimes–related cases were comparatively more likely to end in suspended sentences than in fines (aRRR 0.02, 95% CI 0.01-0.03) or imprisonment without suspension (aRRR 0.55, 95% CI 0.37-0.83; Table 4).

**Table 4:** Exploratory multinomial logistic regression of principal judicial outcomes among prosecuted unlicensed medical practice cases identified from Korean criminal court decisions (2005-2023; n=1532)^a^.

Predictor	Fine, aRRR^b^ (95% CI)^c^	*P* value	Imprisonment without suspension, aRRR (95% CI)^c^	*P* value	Others, aRRR (95% CI)^c^	*P* value
Licensed personnel–only cases (vs ordinary person only)	2.13 (1.36-3.32)	<.001	1.79 (1.02-3.16)	.04	5.14 (2.65-9.99)	<.001
Mixed ordinary person and licensed personnel cases (vs ordinary person only)	3.56 (1.62-7.81)	.002	2.75 (1.18-6.40)	.02	7.43 (2.73-20.21)	<.001
Uncoded (vs ordinary person only)	1.41 (0.53-3.78)	.50	0.74 (0.19-2.93)	.67	2.32 (0.59-9.07)	.23
Act on Special Measures for the Control of Public Health Crimes–related charge involvement (vs not involved)	0.02 (0.01-0.03)	<.001	0.55 (0.37-0.83)	.004	0.09 (0.04-0.18)	<.001
Profit motive (vs no profit)	0.98 (0.57-1.69)	.96	0.57 (0.28-1.13)	.11	0.66 (0.32-1.39)	.28
Korean medical practice (vs general)	0.86 (0.55-1.35)	.51	1.16 (0.77-1.74)	.49	0.93 (0.43-2.02)	.86
Dental practice (vs general)	0.58 (0.30-1.11)	.10	0.49 (0.26-0.92)	.03	0.29 (0.09-1.01)	.05

^a^As the dataset was restricted to detected, prosecuted, and adjudicated unlicensed medical practice cases with at least one conviction, the values estimate associations with ruling type within the prosecuted case set, not the underlying prevalence of unlicensed medical practices, probability of offending, probability of conviction, or guilt. The principal judicial outcome recorded for each case was used as the dependent variable.

^b^aRRR: adjusted relative risk ratio.

^c^The suspended-sentence category was the reference category.

### Secondary Exploratory Analyses of Professional-Related Case Patterns

Table 5 illustrates the secondary exploratory logistic model among 1499 category-coded cases. Professional involvement was less common in Act on Special Measures for the Control of Public Health Crimes–related cases (adjusted odds ratio [aOR] 0.10, 95% CI 0.06-0.14), profit-driven cases (aOR 0.47, 95% CI 0.28-0.77), and Korean medical practice cases (aOR 0.09, 95% CI 0.05-0.16).

In a further exploratory model restricted to 248 cases involving at least one health care professional, physician involvement, compared with involvement of other health care professionals, was associated with health care provider coinvolvement (aOR 4.67, 95% CI 2.40-9.10), a pattern consistent with possible delegation-related or boundary violation scenarios.

**Table 5:** Exploratory logistic regression of health care professional involvement among category-coded prosecuted unlicensed medical practice cases identified from Korean criminal court decisions (2005-2023; n=1499)^a^.

Predictor	aOR^b^ (95% CI)	*P* value
Act on Special Measures for the Control of Public Health Crimes–related charge involvement (vs not involved)	0.10 (0.06-0.14)	<.001
Profit motive (vs no profit)	0.47 (0.28-0.77)	.003
Korean medical practice (vs general)	0.09 (0.05-0.16)	<.001
Dental practice (vs general)	0.55 (0.33-0.93)	.03

^a^This model examined whether a case involved at least one health care professional.

^b^aOR: adjusted odds ratio.

## Discussion

### Principal Findings

This retrospective study used end-to-end neural systems to analyze 1532 criminal precedents and demonstrate the public health utility of legal infoveillance for prosecuted UMPs in South Korea. Three major findings emerged. First, commercially motivated offending predominated, with 91.6% (1404/1532) of cases coded as profit driven. Second, suspended sentences were the most common judicial outcome. Third, UMPs were not confined to ordinary persons without licenses; licensed health care professionals and providers were also substantially represented in the prosecuted case set. Taken together, these findings suggest that UMPs in South Korea should be understood not only as lay “quackery” but also as a problem of regulatory boundary violation within and around formal health care settings.

Health care professionals, particularly physicians, constituted the largest subgroup among them, and fines were common among professional-involved cases. The exploratory inferential analyses further suggested that licensed personnel–only and mixed ordinary person and licensed personnel cases were more likely than ordinary person–only cases to result in fines or imprisonment without suspension rather than suspended sentences. Although judicial records cannot reveal underlying institutional arrangements with certainty, this pattern is consistent with broader concerns about scope-of-practice breaches and commercially driven delegation or task shifting [4,31,32]. In this context, the coinvolvement of physicians and health care providers should be interpreted cautiously as a signal of possible boundary violation scenarios rather than as direct proof of unlawful delegation.

Our findings also help explain why UMPs may persist even in a setting with universal health coverage. In South Korea, the persistence of prosecuted UMPs is unlikely to be explained solely by lack of access to formal care [3]. Rather, the concentration of profit-driven cases and the involvement of licensed actors suggest that legal ambiguity, commercial incentives, and inadequately deterred noncompliance may all contribute to the continuing presence of UMPs [4]. The predominance of suspended sentences and fines may further indicate that many courts viewed these offenses as serious but still compatible with conditional leniency, which may weaken deterrence when the underlying conduct is economically rewarding.

From a methodological standpoint, this study supports legal infoveillance as a supplementary surveillance approach for hidden health-related misconduct. Because UMPs are clandestine and rarely captured directly in routine public health or administrative datasets [19], digitized court decisions provide a rare observational window into detected, investigated, prosecuted, and adjudicated events [18,33]. The framework is potentially transferable to other jurisdictions, but its usefulness will depend on the degree of judicial digitization [14], public access to court records [13], and availability of natural language processing tools capable of handling jurisdiction-specific legal language [34].

The findings need to be carefully interpreted in light of several limitations. Most importantly, the dataset represents prosecuted cases rather than the true prevalence of UMPs. This creates selection and detection bias [33] and makes the observed patterns sensitive to enforcement practices, prosecutorial priorities, and reporting behavior [35]. Accordingly, the results describe the enforcement-visible portion of UMPs rather than the absolute population burden. Therefore, these models should be read only as exploratory descriptions of sentencing patterns among detected, prosecuted, and adjudicated cases; they do not estimate the underlying prevalence of UMPs, the probability that an individual will offend, or the probability that a reported or prosecuted case will result in conviction. Formal interannotator agreement metrics were not calculated, and some complex judicial features had to be simplified into structured variables. Nevertheless, this study narrows an important empirical gap by showing that large-scale legal text analysis can generate population-relevant evidence from otherwise difficult-to-study judicial corpora [24,36].

### Conclusions

This study demonstrates that ML-assisted analysis of criminal court precedents can serve as a useful supplementary method for monitoring hidden UMPs. In South Korea, prosecuted UMPs were predominantly profit driven and involved both ordinary persons and licensed personnel, with exploratory analyses suggesting distinct judicial outcome patterns when health care professionals were involved. These findings support stronger oversight of scope-of-practice and delegation-related violations and reinforce the value of judicial records as a practical source of public health intelligence. Future research should build on this descriptive foundation with richer inferential modeling, improved annotation reliability metrics, and cross-jurisdictional comparisons of how legal design and enforcement shape the detected profile of UMPs.

## Appendix

Multimedia Appendix 1 STROBE checklist.

## Abbreviations

aOR: adjusted odds ratio
aRRR: adjusted relative risk ratio
ML: machine learning
STROBE: Strengthening the Reporting of Observational Studies in Epidemiology
UMP: unlicensed medical practice

## Data Availability

The structured analytic dataset generated during this study is available from the corresponding author on reasonable written request subject to ethical, legal, and data use restrictions. The original court decision corpus obtained through LBox cannot be redistributed by the authors.

## References

[1] Slawomirski L, Klazinga N (2022). The economics of patient safety: from analysis to action. Organisation for Economic Co-operation and Development.

[2] Singh MP, Chand SK, Saha KB, Singh N, Dhiman RC, Sabin LL (2020). Unlicensed medical practitioners in tribal dominated rural areas of central India: bottleneck in malaria elimination. Malar J.

[3] Kwon S (2009). Thirty years of national health insurance in South Korea: lessons for achieving universal health care coverage. Health Policy Plan.

[4] Cho SJ, Yang SR, Ryu S, Park SM (2025). Ghost surgeries in South Korea: variations in practice and the need for clear guidelines in resident surgical training. Korean J Med Ethics.

[5] (2023). Medical Service Act, Act no. 19421. Korea Law Information Center.

[6] (2020). Act on Special Measures for the Control of Public Health Crimes (South Korea), Act No. 17793, Dec 29, 2020. Korean Law Information Center.

[7] (2024). Guidelines for the structure and function of a state medical and osteopathic board. Federation of State Medical Boards.

[8] Dahlawi S, Menezes RG, Khan MA, Waris A, Naseer MM, Saifullah (2021). Medical negligence in healthcare organizations and its impact on patient safety and public health: a bibliometric study. F1000Res.

[9] Wardle J (2014). Holding unregistered health practitioners to account: an analysis of current regulatory and legislative approaches. J Law Med.

[10] Chaudhry HJ, Rhyne J, Cain FE, Young A, Crane M, Bush F (2010). Maintenance of licensure: protecting the public, promoting quality health care. J Med Regul.

[11] Irby DM, Cooke M, OʼBrien BC (2010). The important role of medical licensure in the United States. Acad Med.

[12] Xu B, Hu YN, Zhang Q, Wang H, Wang Z, Yu LY, Cao LP, Yu GP, Hu Y (2016). Prevalence of unlicensed medical practice in China: a retrospective analysis. Lancet.

[13] Jeon HJ, Kim SH, Seo YS (2022). 10 years of civil electronic litigation: achievements and prospects- focusing on civil lawsuits. Judicial Policy Research Institute, Republic of Korea.

[14] Yoon K, Kim MS, Jang J, Jonghyun P, Seonmi J, Nahyun K, Sungpil B (2025). Digitalization of South Korea’s justice system: enhancing access and efficiency. World Bank Group.

[15] Hwang W, Lee D, Cho K, Lee H, Seo M A multi-task benchmark for Korean legal language understanding and judgement prediction. arXiv.

[16] Thomaidou MA, Berryessa CM (2023). Mental illness as a sentencing determinant: a comparative case law analysis based on a machine learning approach. Crim Justice Behav.

[17] Muñoz-Soro JF, del Hoyo Alonso R, Montañes R, Lacueva F (2024). A neural network to identify requests, decisions, and arguments in court rulings on custody. Artif Intell Law.

[18] Legal epidemiology. Centers for Disease Control and Prevention.

[19] Barros JM, Duggan J, Rebholz-Schuhmann D (2020). The application of internet-based sources for public health surveillance (infoveillance): systematic review. J Med Internet Res.

[20] Li J, Xu Q, Cuomo R, Purushothaman V, Mackey T (2020). Data mining and content analysis of the Chinese social media platform Weibo during the early COVID-19 outbreak: retrospective observational infoveillance study. JMIR Public Health Surveill.

[21] LBox Corporation.

[22] von Elm E, Altman DG, Egger M, Pocock SJ, Gøtzsche PC, Vandenbroucke JP (2008). The Strengthening the Reporting of Observational Studies in Epidemiology (STROBE) statement: guidelines for reporting observational studies. J Clin Epidemiol.

[23] Vaswani A, Shazeer N, Parmar N, Uszkoreit J, Jones L, Gomez AN, Kaiser L, Polosukhin I Attention is all you need. arXiv.

[24] Devlin J, Chang MW, Lee K, Toutanova K BERT: pre-training of deep bidirectional transformers for language understanding. arXiv.

[25] Hwang W, Eom S, Lee H, Park HJ, Seo M Data-efficient end-to-end information extraction for statistical legal analysis. arXiv.

[26] Xue L, Constant N, Roberts A, Kale M, Al-Rfou R, Siddhant A, Barua A, Raffel C (2021). mT5: a massively multilingual pre-trained text-to-text transformer. Proceedings of the 2021 Conference of the North American Chapter of the Association for Computational Linguistics: Human Language Technologies.

[27] OpenAI GPT-4 technical report. arXiv.

[28] Parnami A, Lee M Learning from few examples: a summary of approaches to few-shot learning. arXiv.

[29] Sentencing guidelines. Sentencing Commission.

[30] Tkachenko M, Malyuk M, Holmanyuk A, Liubimov N Label Studio: data labeling software. GitHub.

[31] Hooley C, Graaf G, Gopalan G (2021). Scaling up evidence-based treatments in youth behavioral healthcare: social work licensing influences on task-shifting opportunities. Hum Serv Organ Manag Leadersh Gov.

[32] De Maeseneer J, Bourek A, McKee M, Barry M, Brouwer W, Kringos D, Lehtonen L, Murauskiene L, Pita PB, Ricciardi W, Siciliani L (2019). Task shifting and health system design: report of the expert panel on effective ways of investing in health (EXPH). Publications Office of the European Union.

[33] Burris S, Cloud LK, Penn M (2020). The growing field of legal epidemiology. J Public Health Manag Pract.

[34] Ribeiro AL, Araújo OR, Oliveira LB, Inácio M (2022). The Executive Branch decisions in Brazil: a study of administrative decrees through machine learning and network analysis. PLoS One.

[35] Buil-Gil D, Moretti A, Langton SH (2021). The accuracy of crime statistics: assessing the impact of police data bias on geographic crime analysis. J Exp Criminol.

[36] Hong T, Kim D, Ji M, Hwang W, Nam D, Park S (2022). BROS: a pre-trained language model focusing on text and layout for better key information extraction from documents. Proc AAAI Conf Artif Intell.

